# Rescue retrieval of a migrated and fractured biliary plastic stent using a rotatable spiral basket in Roux-en-Y anatomy

**DOI:** 10.1055/a-2839-9747

**Published:** 2026-04-15

**Authors:** Hidenobu Hara, Risa Katsumata, Ami Yoshinaga, Harujiro Yamamoto, Shiori Ito, Kouhei Yoshino, Shinya Sakita

**Affiliations:** 1 53327 Department of Gastroenterology, Yokohama City Minato Red Cross Hospital, Yokohama, Japan

Intraductal stent migration and retained fragments after stent fracture can be difficult to retrieve endoscopically. Rescue retrievals using lithotripsy devices have been reported 1 2 . Other extraction techniques include cholangioscopy-directed snaring or grasping and fluoroscopy-guided retrieval using snares or conventional baskets. A rotatable spiral basket catheter allows clockwise and counterclockwise rotations and standard to-and-fro manipulation, and its utility for foreign body removal has been described ( 3 4 5 , Fig. 1 ).

**Fig. 1:**
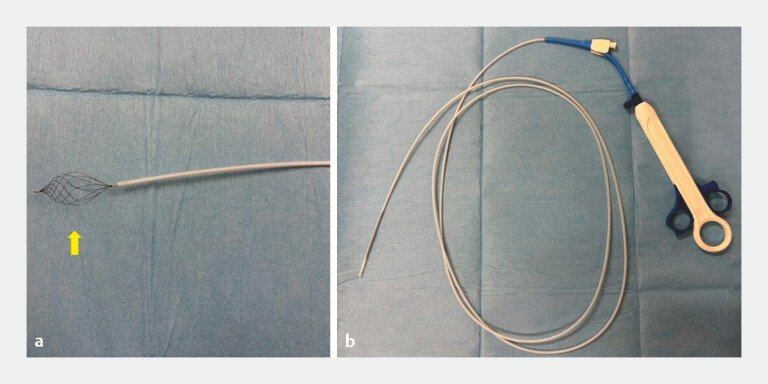
The structure of the rotatable spiral basket catheter. **a** Distal basket tip with an 8-wire spiral design (arrow); basket length, 40 mm; basket diameter, 20 mm. **b** An overall view of the device and the handle, which allows clockwise and counterclockwise rotations; working length, 200 cm.

An 84-year-old woman with Roux-en-Y reconstruction for gastric cancer underwent biliary drainage for acute cholangitis secondary to common bile duct stones, with the placement of two plastic stents (7-Fr, 12-cm and 7-Fr, 7-cm). After stent migration and fracture were identified, she underwent enteroscopy-assisted endoscopic retrograde cholangiopancreatography (ERCP; Video 1 ).

Rotatable spiral basket rescue for a migrated biliary stent and a fractured fragment in Roux-en-Y anatomy.Video 1

Enteroscopy-assisted ERCP using a short-type single-balloon enteroscope (SIF-H290S; Olympus, Tokyo, Japan) revealed the complete intraductal migration of the 7-Fr, 12-cm stent ( Fig. 2 **a, b** ). The 7-Fr, 7-cm stent had fractured on the hepatic side. The detached fragment was floating within the common bile duct ( Fig. 2 **a** ). Endoscopic papillary balloon dilation (8 mm) was performed ( Fig. 3 **a** ). Balloon sweeping was also unsuccessful owing to pronounced ductal angulation. Neither the migrated stent nor the fractured fragment could be captured ( Fig. 3 **b** ). Retrieval using grasping forceps failed because marked ductal dilatation prevented stable grasping ( Fig. 3 **c** ).

**Fig. 2:**
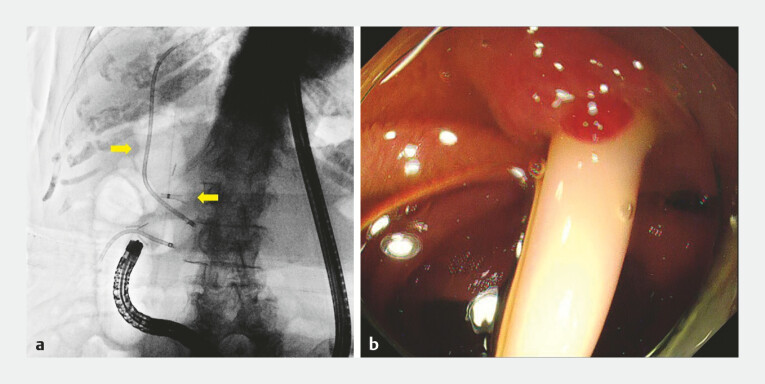
Pre-procedural fluoroscopic and endoscopic findings. **a** Fluoroscopy shows the complete intraductal migration of a 7-Fr, 12-cm plastic biliary stent and a free-floating intraductal fragment from a fractured 7-Fr, 7-cm stent (arrows). **b** An endoscopic view at the papilla shows only one stent.

**Fig. 3:**
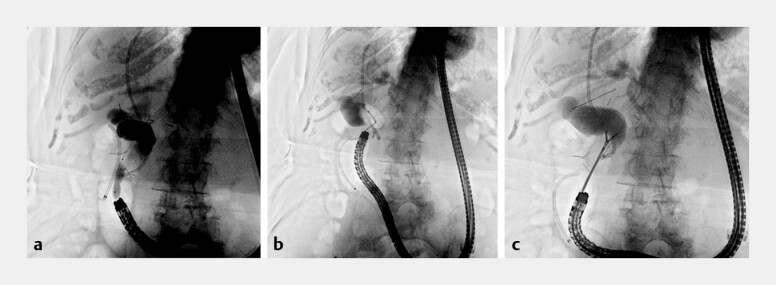
Papillary dilation and failed retrieval attempts using conventional devices. **a** Endoscopic papillary balloon dilation was performed with an 8-mm balloon catheter. **b** Balloon sweeping was unsuccessful because of marked ductal angulation. **c** Retrieval using grasping forceps was attempted for the migrated stent and the fractured fragment but was unsuccessful.

A rotatable spiral basket catheter was then deployed within the bile duct. With several clockwise and counterclockwise rotations, the migrated stent was entangled within the spiral basket and removed ( Fig. 4 **a** ). The floating fractured fragment was subsequently guided into and secured within the spiral basket using the same maneuver, enabling complete retrieval ( Fig. 4 **b, c** ). No procedure-related adverse events occurred.

**Fig. 4:**
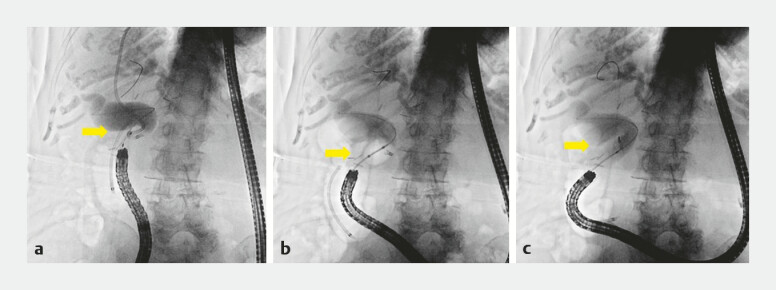
Fluoroscopic images of failed balloon retrieval and rescue retrieval using a rotatable spiral basket catheter. **a** The migrated 7-Fr, 12-cm stent was readily removed after several rotational maneuvers (arrow). **b** Balloon sweeping was also unsuccessful (arrow). **c** With additional rotational maneuvers, the fragment was guided into and secured within the spiral basket, enabling retrieval (arrow).

This case illustrates that a rotatable spiral basket catheter can serve as a useful rescue option in a markedly dilated and angulated bile duct during enteroscopy-assisted ERCP in surgically altered anatomy by capturing targets through an “entanglement” mechanism rather than direct grasping.

Endoscopy_UCTN_Code_TTT_1AR_2AZ

## References

[1] Ohno A Fujimori N Hirahata K The novel basket catheter for retrieval of a migrated biliary inside stent Endoscopy 2022 54 E596 E597 10.1055/a-1711-4161 34933370

[2] Sumi K Kato H Okada Y Retrieval of a migrated biliary stent using a customized goose-neck snare Endoscopy 2025 57 E1063 E1064 10.1055/a-2686-3583 40935143 PMC12425594

[3] Koga T Matsumoto K Ko YL Tornado cleaning technique for food impaction in gastroduodenal stent using a high-rotational spiral basket catheter Endoscopy 2025 57 E889 E890 10.1055/a-2658-0539 40829747 PMC12364554

[4] Ishikawa S Koizumi M Kokubu M Retrieval technique for a sheared guidewire remnant in the gallbladder duct using a novel basket catheter Endoscopy 2024 56 E939 E940 10.1055/a-2432-3302 39515761 PMC11548961

[5] Watanabe M Okuwaki K Kusano C Stent retrieval technique using a basket catheter with a rotation function for retrieval of thread-attached stent Dig Endosc 2025 37 1377 1378 10.1111/den.70013 40776846

